# Supplementary material for: The role of the PZP domain of AF10 in acute leukemia driven by AF10 translocations
Source: Nat Commun. 2021 Jul 5;12:4130. doi: 10.1038/s41467-021-24418-9 (PMC8257627; doi:10.1038/s41467-021-24418-9)
Supplement: Supplementary file 1 — Supplementary Info [file 41467_2021_24418_MOESM1_ESM.pdf]

Supplementary Information

The role of the PZP domain of AF10 in acute leukemia driven by AF10 translocations

Brianna J. Klein, et al.

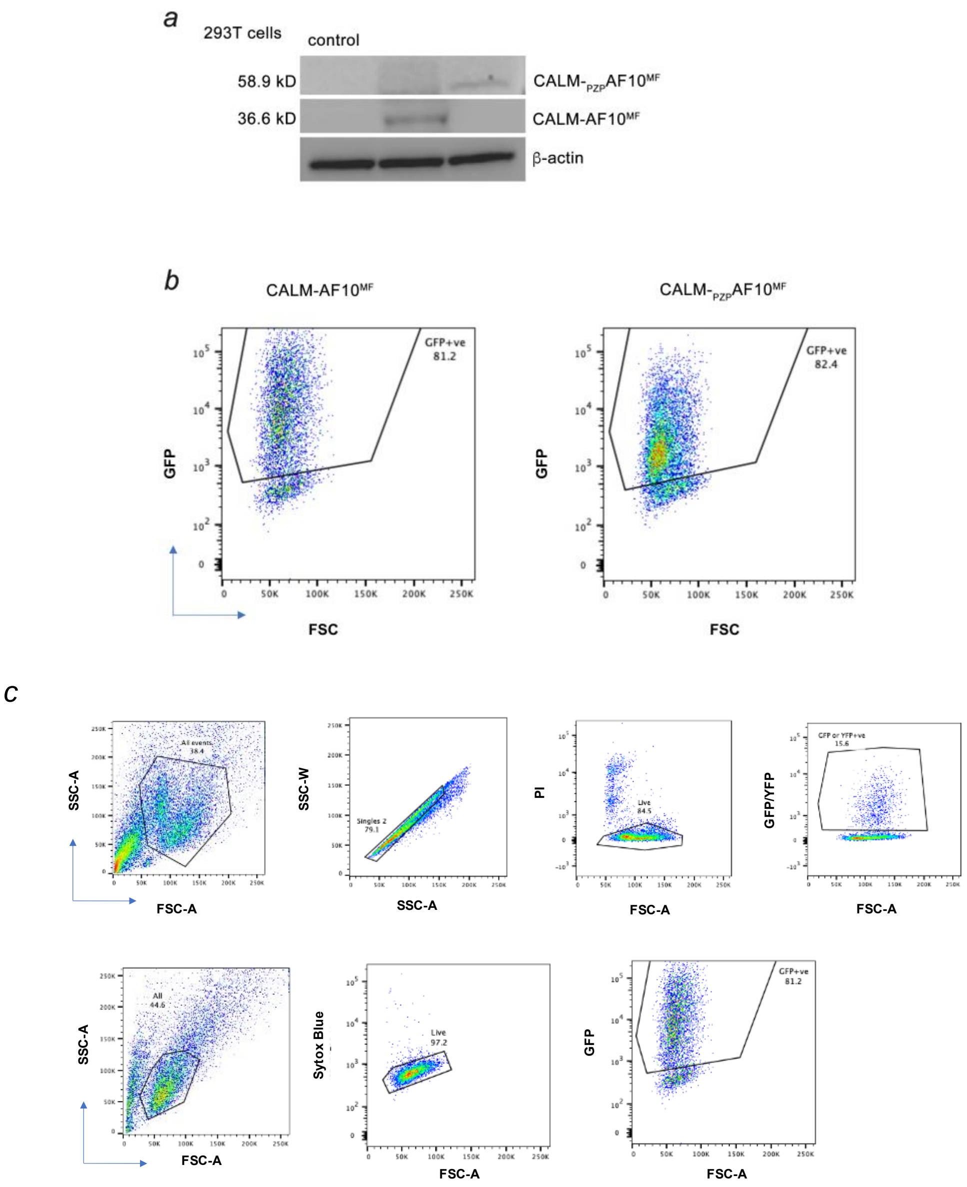

Supplementary Figure 1. Expression of CALM-AF10^{MF} and CALM-$_{PZP}$AF10^{MF} proteins. (a) 293T cells transfected with 1XFLAG CALM-AF10^{MF} or 1XFLAG CALM-$_{PZP}$AF10^{MF} constructs were analyzed by immunoblotting for indicated proteins. Non transfected cells were used as control. (b) 293T cells transfected with CALM-AF10^{MF} or CALM-$_{PZP}$AF10^{MF} constructs were analyzed for Green Fluorescent Protein (GFP) +ve in flow cytometry. (c) Schematics of FACS gating: a representative example of gating used for sorting CALM-AF10^{MF} or CALM-$_{PZP}$AF10^{MF} transformed murine HSPCs (top). Gating strategy to analyse expression levels as well as percentage of GFP positive cells expressing CALM-AF10^{MF} and CALM-$_{PZP}$AF10^{MF} in 293T (bottom). Related to Figures 1 and 2.

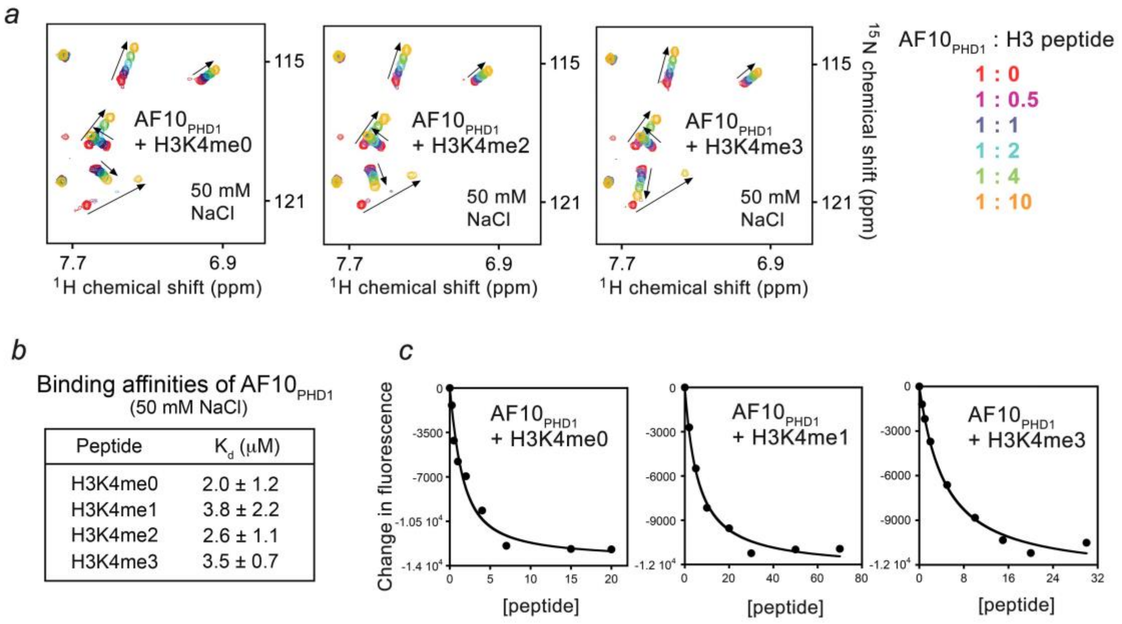

Supplementary Figure 2. (a) Overlay of $^{1}H,^{15}N$ HSQC spectra of $AF10_{PHD1}$ in the presence of increasing amounts of $H3_{1-12}$, $H3K4me2_{1-12}$ and $H3K4me3_{1-12}$ peptides in low salt (50 mM NaCl) buffer. Spectra are colored according to the protein:peptide molar ratio. (b, c) Binding affinities (b) and binding curves (c) used to determine K_d values in low salt (50 mM NaCl) buffer by tryptophan fluorescence. K_ds are represented as mean values +/- S.D. from three independent experiments (n=3). Related to Figure 3.

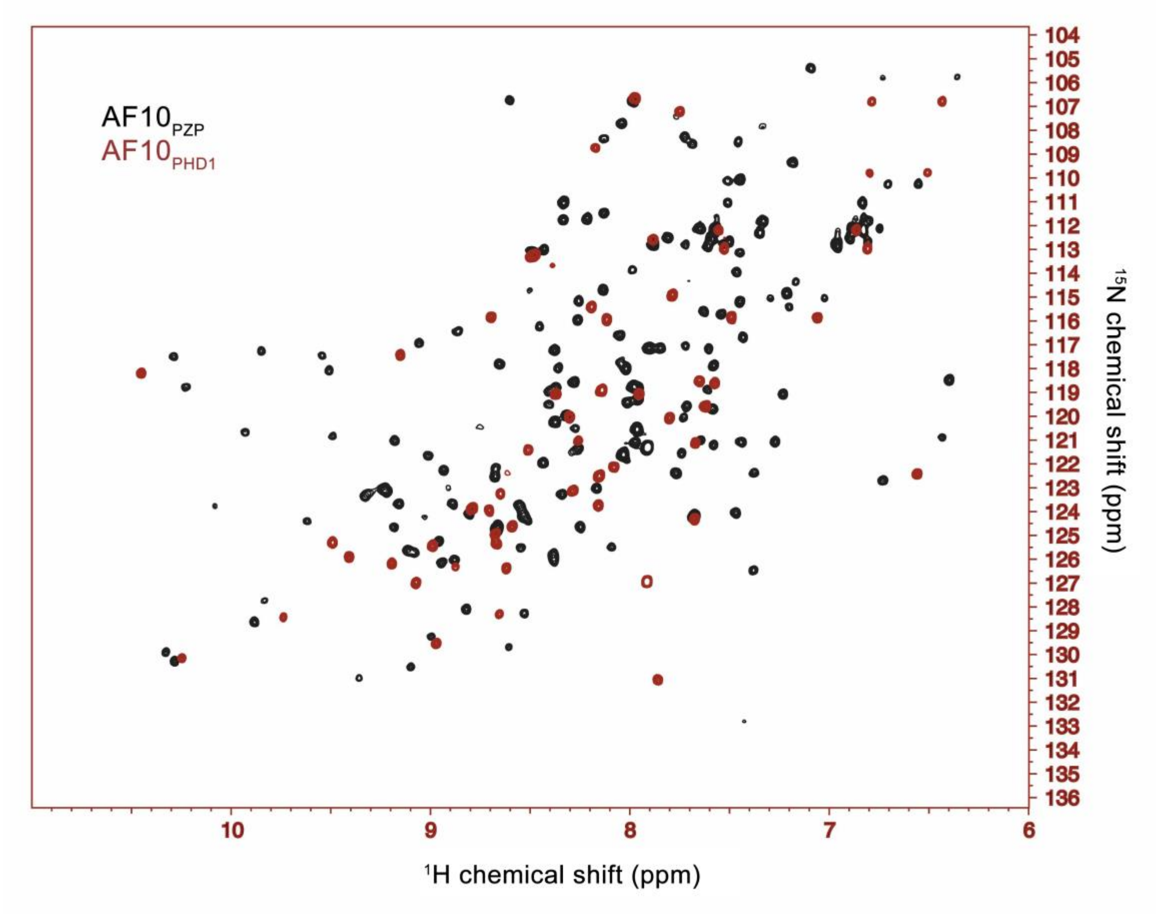

Supplementary Figure 3. Superimposed ^{1}H,^{15}N HSQC spectra of $AF10_{PHD1}$ and $AF10_{PZP}$ in apo- states. Related to Figure 3.

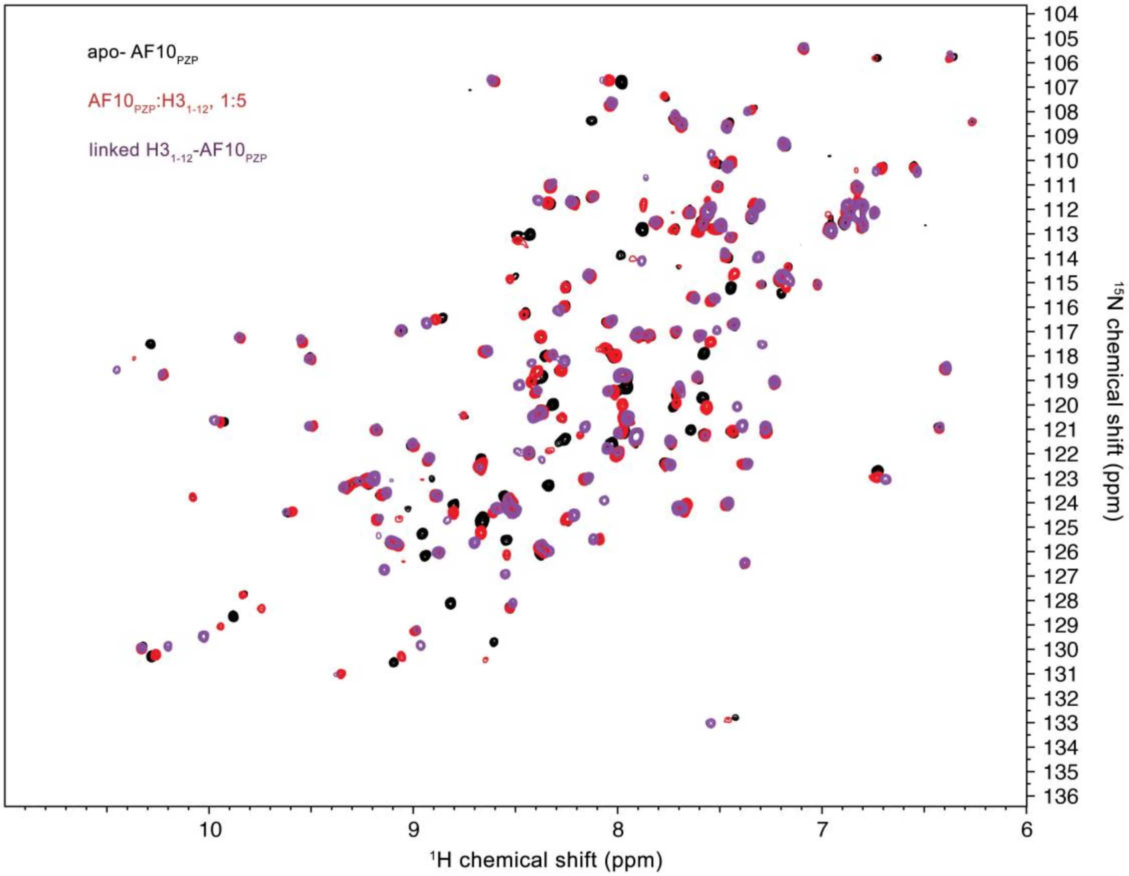

Supplementary Figure 4. Superimposed $^{1}H,^{15}N$ HSQC spectra of the linked $H3_{1\text{-}12}$-$AF10_{PZP}$ construct (purple), the isolated $AF10_{PZP}$ in the presence of a 5-fold excess of $H3_{1\text{-}12}$ peptide (red) or the apo- state of $AF10_{PZP}$ (black). Related to Figure 4.

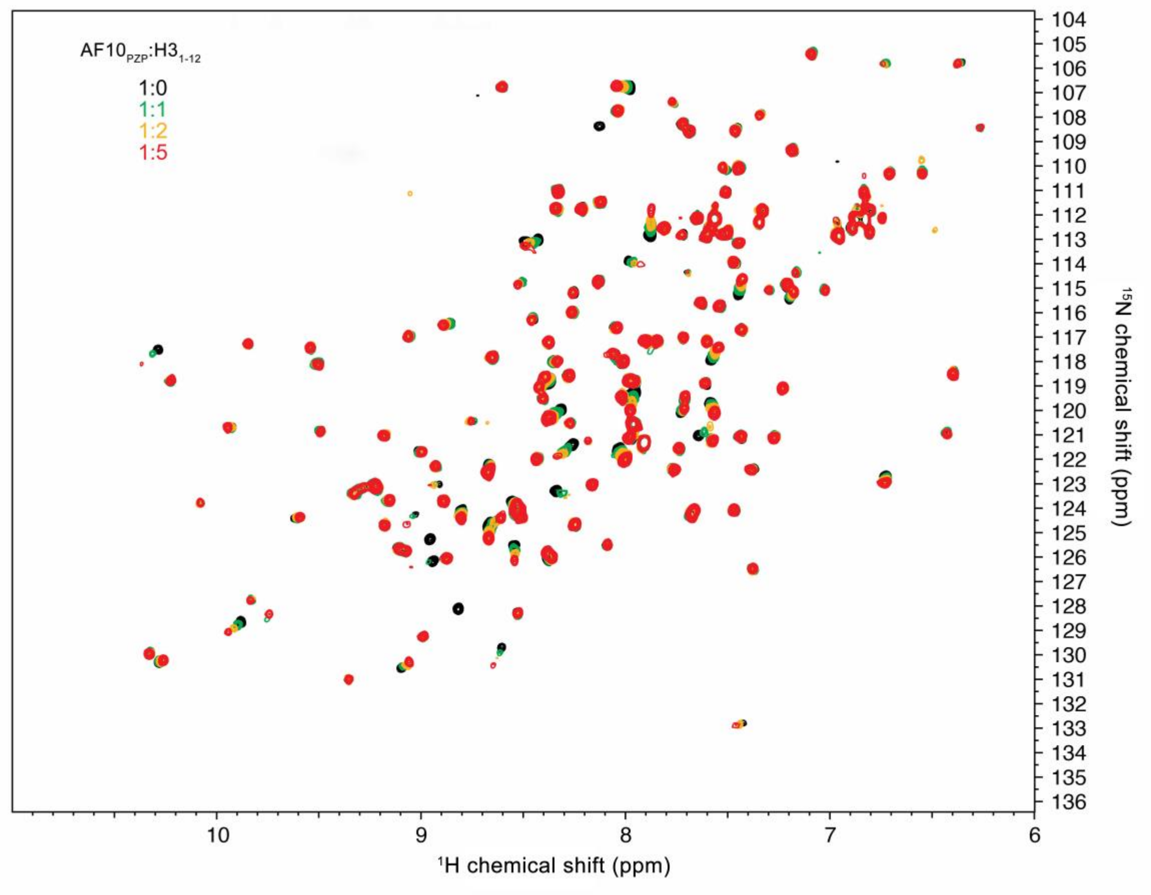

Supplementary Figure 5. Superimposed ^{1}H,^{15}N HSQC spectra of AF10$_{PZP}$ collected upon titration with the indicated H3 peptide. Spectra are color coded according to the protein:peptide molar ratio. Related to Figure 5.

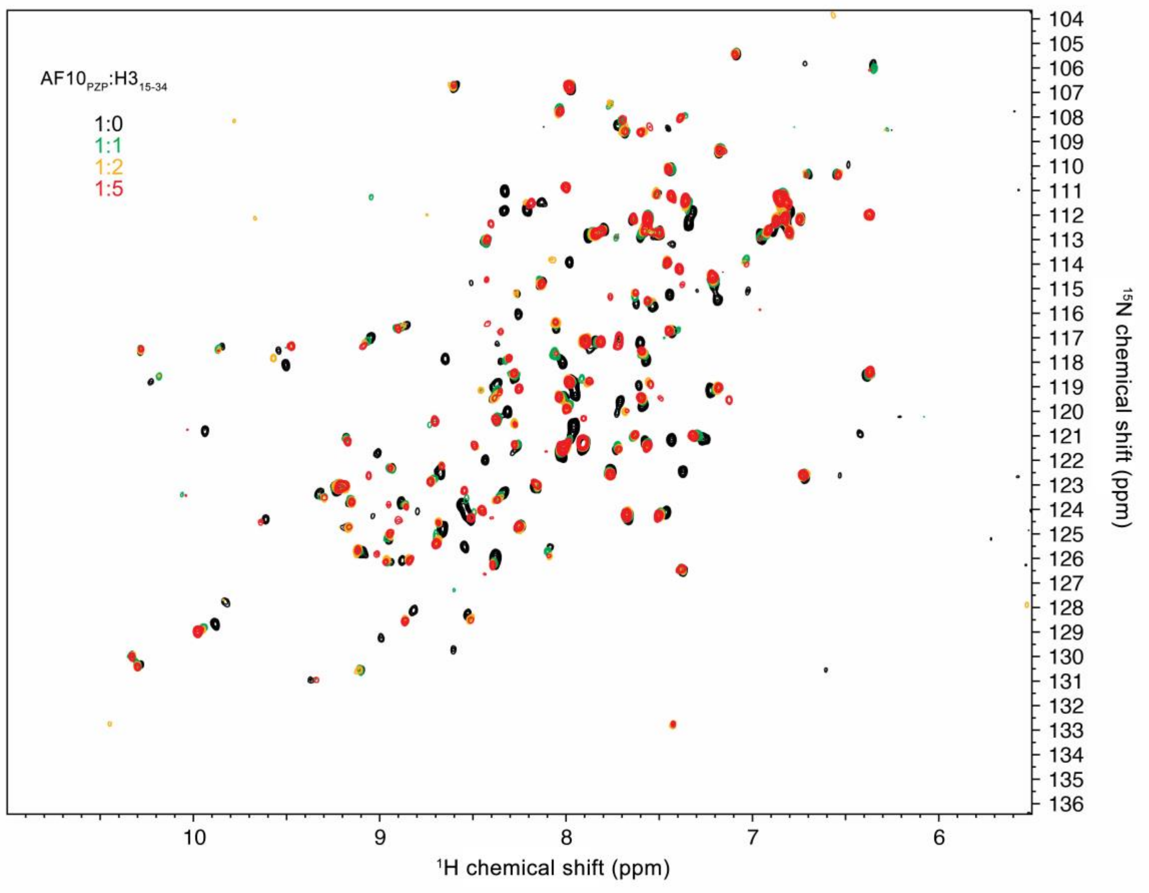

Supplementary Figure 6. Superimposed $^{1}H,^{15}N$ HSQC spectra of $AF10_{PZP}$ collected upon titration with the indicated H3 peptide. Spectra are color coded according to the protein:peptide molar ratio. Related to Figure 5.

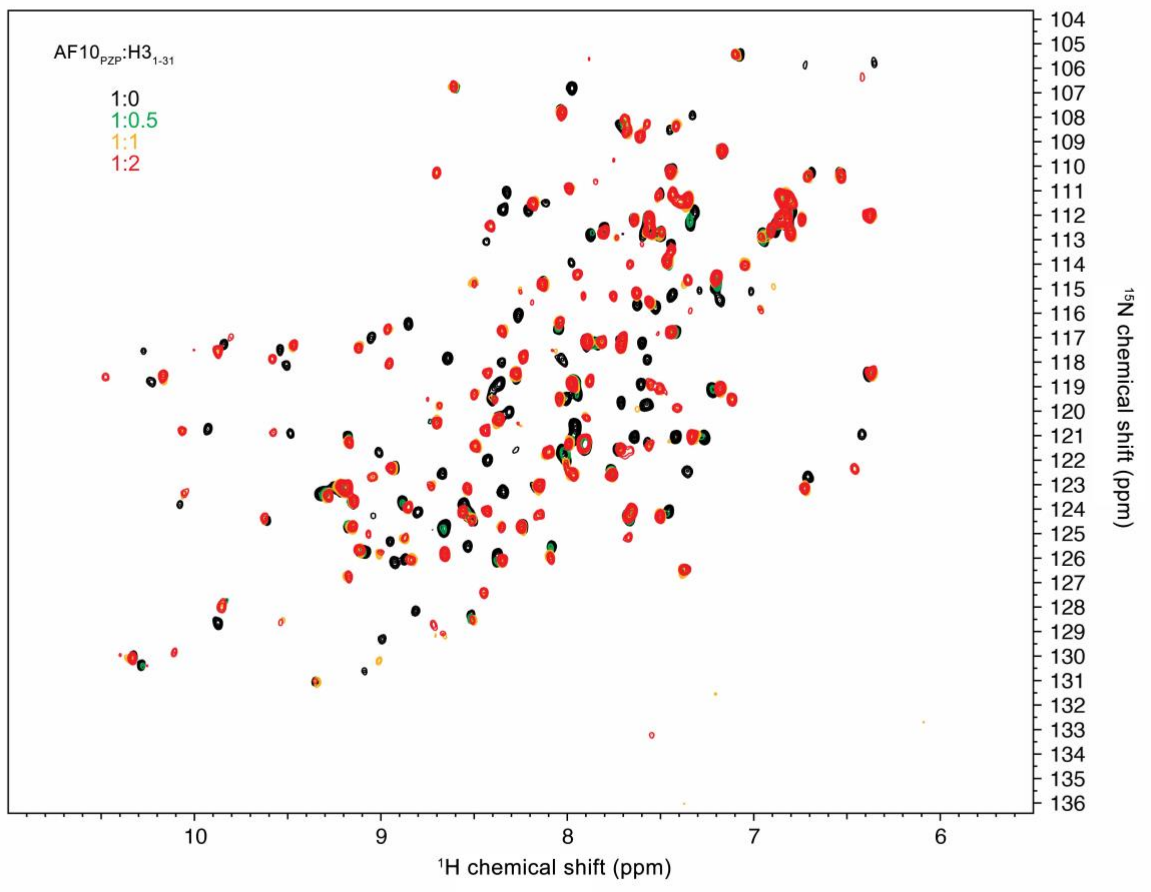

Supplementary Figure 7. Superimposed $^{1}H,^{15}N$ HSQC spectra of $AF10_{PZP}$ collected upon titration with the indicated H3 peptide. Spectra are color coded according to the protein:peptide molar ratio. Related to Figure 5.

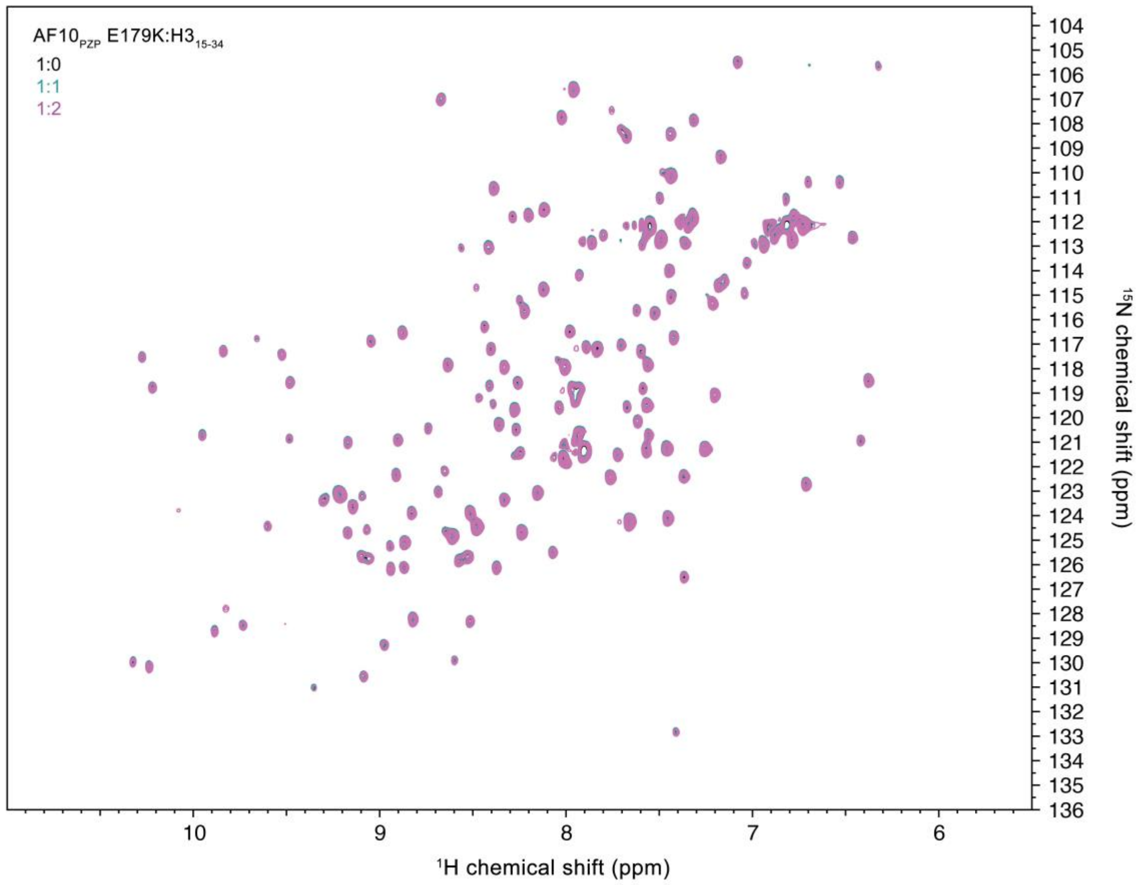

Supplementary Figure 8. Superimposed $^{1}H,^{15}N$ HSQC spectra of the mutated $AF10_{PZP}$ collected upon titration with the indicated H3 peptide. Spectra are color coded according to the protein:peptide molar ratio. Related to Figure 6.

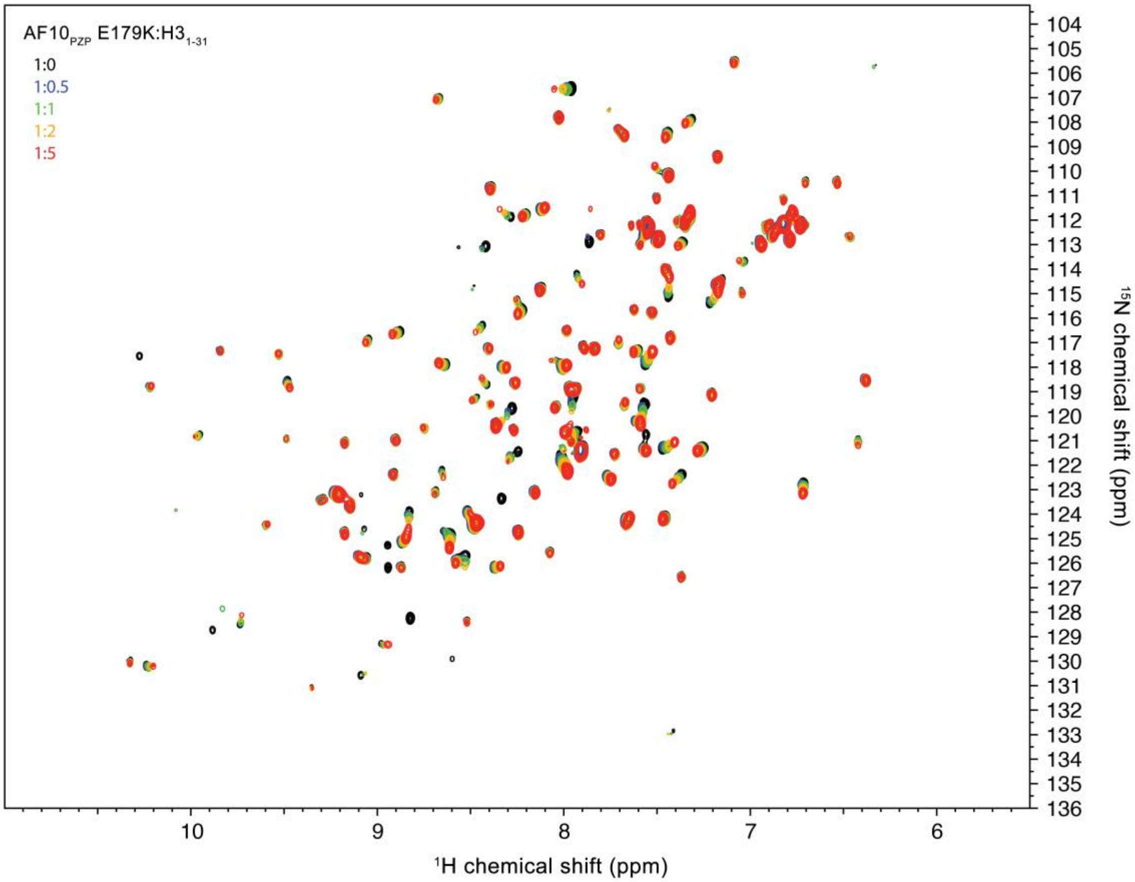

Supplementary Figure 9. Superimposed ^{1}H,^{15}N HSQC spectra of the mutated AF10$_{PZP}$ collected upon titration with the indicated H3 peptide. Spectra are color coded according to the protein:peptide molar ratio. Related to Figure 6.

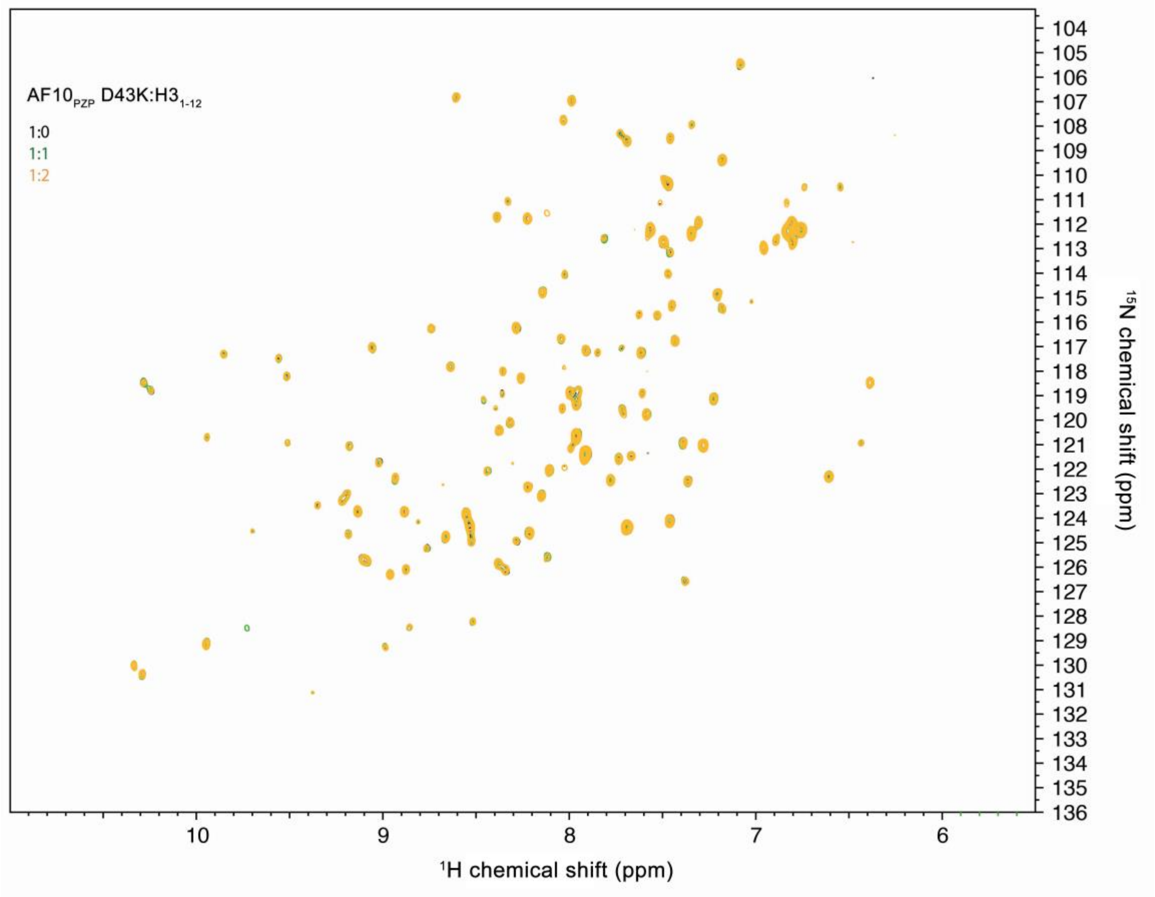

Supplementary Figure 10. Superimposed $^{1}H,^{15}N$ HSQC spectra of the mutated $AF10_{PZP}$ collected upon titration with the indicated H3 peptide. Spectra are color coded according to the protein:peptide molar ratio. Related to Figure 6.

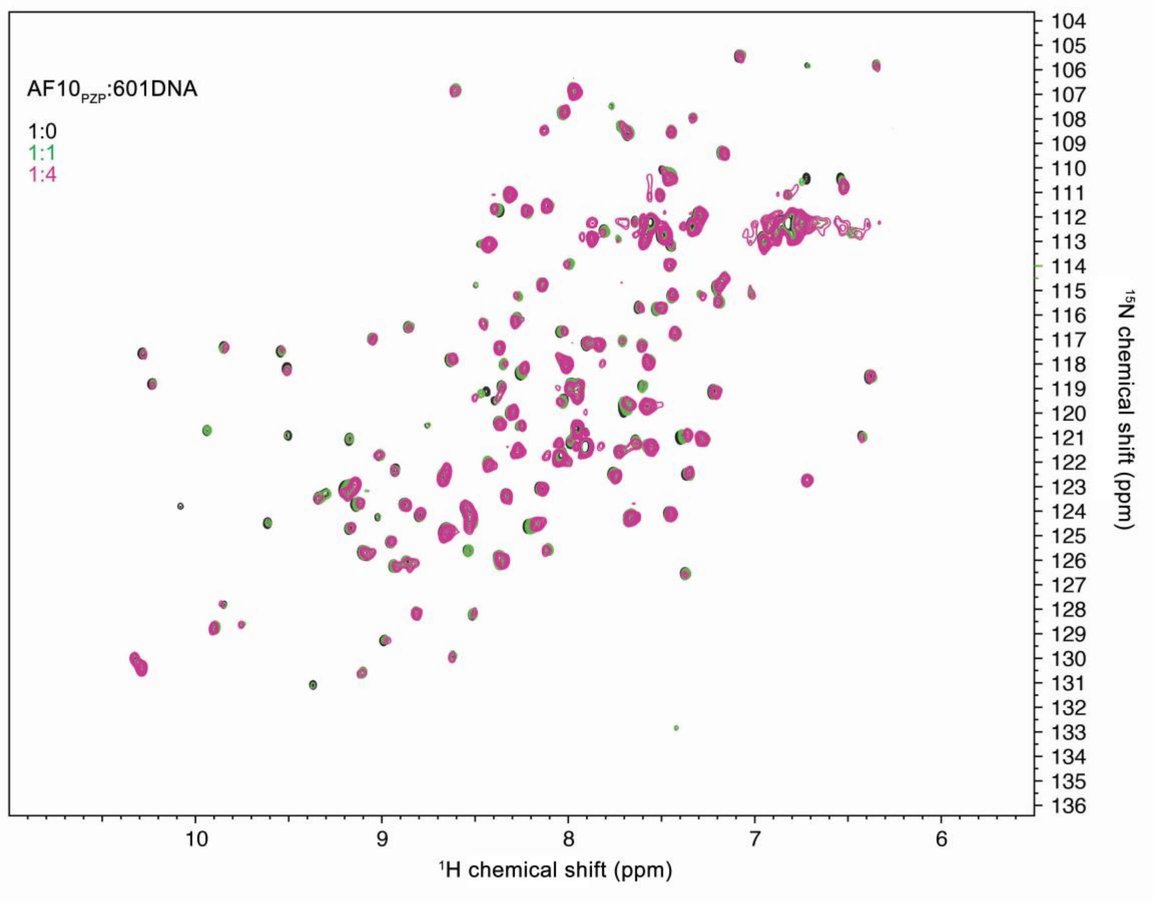

Supplementary Figure 11. Overlay of $^{1}H,^{15}N$ HSQC spectra of $AF10_{PZP}$ in the presence of increasing amount of 147 bp 601 DNA. Spectra are colored according to the protein:DNA molar ratio. Related to Figure 7.

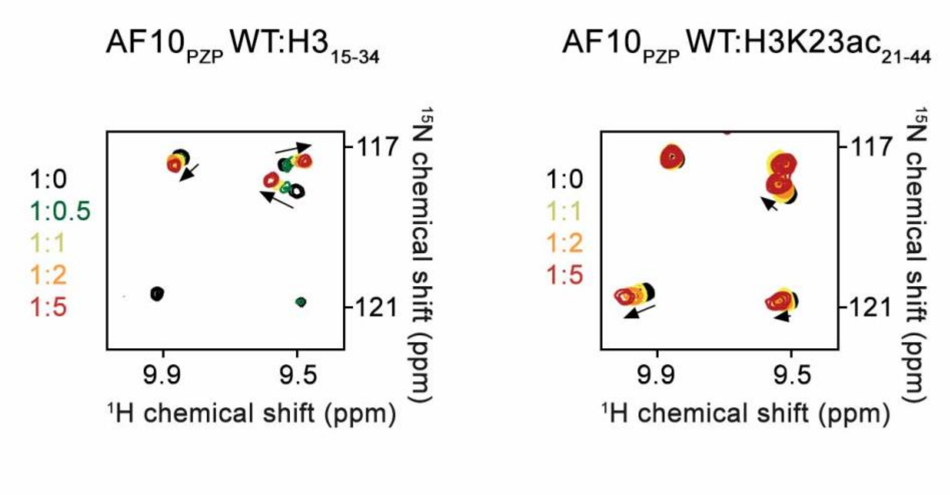

Supplementary Figure 12. Superimposed ^{1}H,^{15}N HSQC spectra of AF10$_{PZP}$ collected upon titration with the indicated H3 peptides. Spectra are color coded according to the protein:peptide molar ratio. Related to Figure 7.

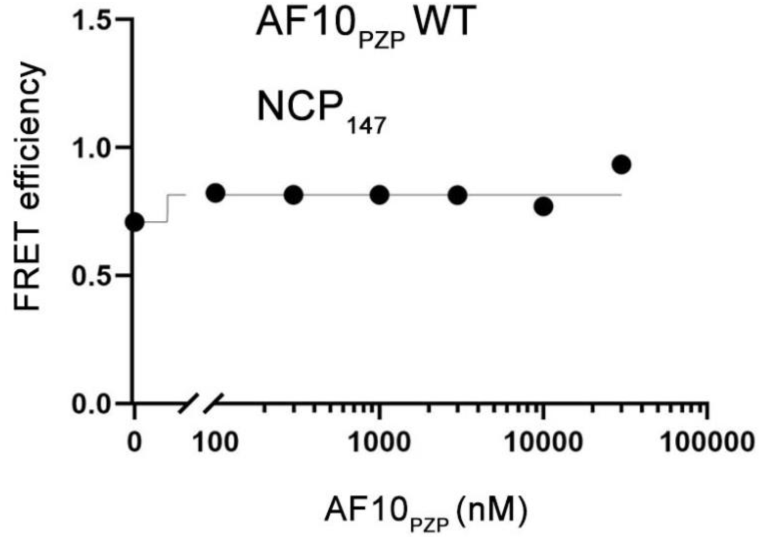

Supplementary Figure 13. FRET efficiency of the Cy3-Cy5 labeled NCP_{147} upon addition of $AF10_{PZP}$. Data are represented as mean values +/- S.D. from three independent experiments (n=3). Related to Figure 7.

Supplementary Table 1. Data collection and refinement statistics

	$AF10_{PZP}$:$H3_{1\text{-}12}$
**Data collection**	
Space group	P 1 21 1
Cell dimensions	
a, *b*, *c* (Å)	42.9, 54.3, 47.8
α, β, γ (°)	90.0, 106.8, 90.0
Resolution (Å)	41.08-2.10(2.16-2.10) *
R_{pim}	0.042(0.415)
$I / \sigma I$	15.9(1.7)
Completeness (%)	97.9(83.3)
Redundancy	13.2(8.8)
**Refinement**	
Resolution (Å)	41.08-2.10
No. reflections	12121
R_{work} / R_{free}	0.1646/0.2059
No. atoms	1599
$AF10_{PZP}$	1353
H3	90
Zn	5
Water	151
B-factors	35.58
$AF10_{PZP}$	34.96
H3	33.72
Zn	25.78
Water	42.61
R.m.s. deviations	
Bond lengths (Å)	0.007
Bond angles (°)	0.828
Ramachandran outliers	0
Ramachandran allowed (%)	0.56
Ramachandran favored (%)	99.44

*Values in parentheses are for highest-resolution shell.
